# Transfer of Maternal Immunity to Newborns of Diabetic Mothers

**DOI:** 10.1155/2012/928187

**Published:** 2012-09-09

**Authors:** Eduardo Luzía França, Iracema de Mattos Paranhos Calderon, Elisa Lima Vieira, Glilciane Morceli, Adenilda Cristina Honorio-França

**Affiliations:** ^1^Institute of Biological and Health Science, Federal University of Mato Grosso, 78600-000 Barra do Garças, MT, Brazil; ^2^Postgraduate Program in Gynecology, Obstetrics and Mastology, Botucatu Medical School, Sao Paulo State University (Unesp), 18600-000 Sao Paulo, SP, Brazil

## Abstract

This study was carried out with hyperglycemic pregnant women to investigate the transfer of antibody classes to newborns across the placenta or by colostrum and the functional activity of phagocytes in maternal blood, cord blood, and colostrum from diabetes mothers. Samples from maternal blood, cord blood, and colostrum were collected from 20 normoglycemic and 20 hyperglycemic pregnant women. We determined antibodies levels, superoxide release, phagocytosis and bactericidal activity of phagocytes. We demonstrated that IgG levels in cord blood were higher in the hyperglycemic group. IgA and IgM levels were higher in maternal than in cord blood samples. Plasma antibody levels were lower in hyper- than in normoglycemic women. The colostrum of diabetic mothers had lower IgA and IgG levels. Colostrum and maternal blood phagocytes when exposed to EPEC increased the superoxide release. Cord blood phagocytes of hyperglycemic group, independently of bacteria, had higher superoxide release. Colostrum and blood phagocytes from diabetic group exhibited some phagocytic and microbicidal activity in response to EPEC. Mononuclear phagocytes from cord blood had the lowest phagocytosis, and bactericidal activity for EPEC, regardless of glycemic status. These data showed that hyperglycemia altered IgG transfer across the placenta and decreases immunoglobulin levels in maternal blood and colostrum.

## 1. Introduction

Antibodies and resistance factors of the amniotic fluid, with anti-infectious activity [1, 2], are transmitted to the fetus via the placenta. The passive transfer of IgG antibodies across the placenta compensates for low antibody production during intrauterine life [3, 4]. This passive immunity acquired by the fetus is crucial for the adaptation of the newborn to the extrauterine environment, providing protection against infectious agents during the first months of life. The maternal-fetal transfer of antibodies appears to be an active mechanism linked to specific characteristics of the IgG Fc fragment [1, 5]. With the transfer of IgG across the placenta, the mother transmits her immune experience to the fetus. This is particularly important since the mother develops defense factors that are specific for microorganisms occurring in the same environment the child will likely occupy.

At birth, the newborn is suddenly transferred from a sterile environment to an environment populated by microorganisms, where it becomes exposed to different microbial agents, including some with pathogenic potential. Newborns have an immature immune system, and to address this deficiency, especially at the mucosal level, nature has developed an immune supplement that is provided by colostrum and breast milk, which are extremely rich in defense factors [6]. Both colostrum and mature milk contain a large variety of nutrition factors as well as soluble and cellular components involved in anti-infective immune protection [7–9].

Breastfeeding may modify the natural development of type I diabetes because it protects children against a number of viral diseases, avoiding or delaying disease onset [10]. Furthermore, breast milk compensates for inadequate immunoglobulin production and protects the child against gastrointestinal and respiratory tract infections [11, 12]. 

Cells with phagocytic and microbicidal activity are some of the multiple immune components of colostrum and mature milk that play an important role in child protection [8, 9]. Some studies report that diabetic patients have reduced phagocytic activity, low microbicidal activity, and reactive oxygen species production due to changes in antioxidant systems. The reduction in phagocytic and leukocyte microbicidal activity is likely related to an increase in blood glucose levels [13, 14]. 

With the advances in the metabolic control of glucose, diabetic women are more likely to become pregnant. However, due to alterations in glucose metabolism, the components in the milk they produce are changed. To the best of our knowledge, there are no studies on the functional activity in the blood and colostrum of diabetic mothers as well as on the transfer of specific antibody classes to the newborn. The present study shows the transfer of antibody classes across the placenta or by colostrum and the functional activity of phagocytes in maternal blood, cord blood, and colostrum of diabetic women. 

## 2. Methods

### 2.1. Subject Evaluation

This cross-sectional study evaluated 40 diabetic and normoglycemic women (18 to 35 years of age) treated at the Diabetes and Pregnancy Service of the Botucatu Medical School Obstetric Course, UNESP, Botucatu, SP. All the volunteers signed an informed consent form and the research was approved by the local Research Ethics Committee. 

The pregnant women selected during prenatal care were divided into two groups with different glycemic status, as determined by the oral glucose tolerance test (g-OGTT 100 g) and the glycemic profile (GP) [15]. The normoglycemic group (*N* = 20) had normal GP, whereas the hyperglycemic group (*N* = 20) was diagnosed with clinical diabetes, abnormal prepregnancy, and insulin-dependence. The subjects continued treatment at the facility irrespective of diagnosis, and the hyperglycemic patients followed a specific glycemic control protocol [15]. The variables controlled in both groups during pregnancy were smoking status (yes/no), arterial hypertension (yes/no), and glycemic index (GI), which was the mean plasma glucose level measured over the gestation. GI was classified as adequate (GI < 120 mg/dL) or inadequate (GI ≥ 120 mg/dL) [15]. 

The normoglycemic women gave birth at 38 ± 0.8 weeks gestation and the hyperglycemic at 37 ± 0.2 weeks. Newborn weight was 2830.3 ± 321.7 g in the normoglycemic group and the 3193.5 ± 254.2 g in the hyperglycemic group. Immunological tests were carried out using samples of maternal blood, cord blood and colostrum from both experimental groups.

### 2.2. Colostrum Sampling

We collected 15 mL of colostrum 48 to 72 h postpartum. Samples were centrifuged at 160 G and 4°C for 10 min. The upper fat layer was discarded, cells were separated, and the aqueous supernatant stored at −80°C for later antibody analysis.

### 2.3. Blood Sampling

Samples of 15 mL of maternal blood and cord blood were collected at birth in tubes with anticoagulant. We centrifuged them at 160 G for 15 min to separate plasma from the cells. These cells were used immediately and the plasma was stored at −80°C for later antibody analysis. 

### 2.4. Immunoglobulin Determination

Colostrum and plasma IgA, IgG and IgM levels were determined by quantitative radial immunodiffusion (RID) according to Mancini et al. [16]. A tube containing 10 mL of 1% agarose was heated to fusion in water-bath and transferred to another bath at 56°C for temperature stabilization. Anti-human IgA, lamb serum (Biolab, São Paulo, Brazil), anti-human IgM (Sigma, St. Louis, USA), and anti-human IgG (Sigma, St. Louis, USA) antibodies were added to the tubes and mixed to the agarose by tube inversion. The mixture was placed between two glass plates separated by a spacer. After solidification, the plates were perforated and the samples applied. Antibody content in the colostrum samples was determined using the Kallestad standard curve.

### 2.5. Colostral Phagocyte Separation

The colostrum samples were centrifuged at 160 G and 4°C for 10 min and separated into three phases: cell pellet, an intermediate aqueous phase, and a lipid-containing supernatant [7]. The cells were separated by a Ficoll-Paque gradient (Pharmacia, Upsala, Sweden), producing preparations with 98% pure mononuclear cells that were analyzed using light microscopy. The resulting MN phagocyte suspensions were adjusted to a concentration of 2 × 10^6^ cells/mL.

### 2.6. Blood MN Phagocyte Separation

Maternal blood and cord blood samples were fractionated over a Ficoll-Paque (Pharmacia, Uppsala Sweden) density gradient (density 1.077 g/L). Mononuclear (MN) cells were separated and resuspended independently in serum-free medium 199. MN phagocytes were washed separately twice in serum-free medium 199. This procedure yielded 95% pure MN preparations as analyzed by light microscopy using the trypan blue exclusion assay. The resulting MN phagocyte suspensions were adjusted to a concentration of 2 × 10^6^ cells/mL. 

### 2.7. Release of Superoxide Anion

Superoxide release was determined by cytochrome C (Sigma, ST Loius, USA) reduction [10, 17]. Briefly, mononuclear phagocytes (blood and colostrum) and bacteria were mixed and incubated for 30 min for phagocytosis. Cells were then resuspended in PBS containing 2.6 mM CaCl_2_, 2 mM MgCl_2_, and cytochrome C (Sigma, ST Loius, USA; 2 mg/mL). The suspensions (100 *μ*L) were incubated for 60 min at 37°C on culture plates. The reaction rates were measured by absorbance at 550 nm and the results were expressed as nmol/O_2_^−^. All the experiments were performed in duplicate.

### 2.8. Bactericidal Assay

We used enteropahtogenic *Escherichia coli* (EPEC) to assess the functional activity of blood and colostrum phagocytes. This bacterium was isolated from stools of an infant with acute diarrhea (serotype 0111:H2, LA1, eae1, EAF1, bfp1) [18]. The material was prepared and adjusted to 10^8^ bacteria/mL, as described by Honorio-França et al. [7].

Microbicidal activity and phagocytosis were evaluated by the acridine orange method described by Bellinati-Pires et al. [19]. Equal volumes of bacteria and cell suspension were mixed and incubated at 37°C for 30 min under continuous shaking. Phagocytosis was stopped by cooling the test tubes in ice. To eliminate extracellular bacteria, suspensions were centrifuged twice (160 ×g, 10 min, 4°C). Cells were resuspended in serum-free medium 199 and centrifuged. The supernatant was discarded and the sediment dyed with 200 *μ*L of acridine orange (14.4 g/L) for 1 min. The sediment was resuspended in cold culture 199, washed twice, and observed under immunofluorescence microscope at 400x and 1000x magnification. The phagocytosis index was calculated by counting the number of cells ingesting at least 3 bacteria in a pool of 100 cells. To determine the bactericidal index, we stained the slides with acridine orange and counted 100 cells with phagocytized bacteria. The bactericidal index corresponds to the ratio between orange-stained (dead) and green-stained (alive) bacteria × 100 [9]. The experiments were performed in duplicate or triplicate. 

### 2.9. Statistical Analysis

Analysis of variance (ANOVA) was used to evaluate antibody class, superoxide, phagocytosis, and bactericidal index for the EPEC strain. Statistical significance was considered for a  *P*  value less than 0.05.

## 3. Results

Glucose levels in colostrum, maternal blood, and cord blood were higher in the hyper- than in the normoglycemic women (Table 1). Leukocyte retrieval and viability in colostrum samples were similar between the groups, but leukocyte count was lower in cord blood than in maternal blood and colostrum, irrespective of glycemic status (Table 1).

Compared to the normoglycemic mothers, the hyperglycemic women had lower IgA and IgG levels in colostrum and lower IgG and IgM levels in blood. IgA levels in maternal blood did not vary between the groups. IgA was predominantly found in colostrum, whereas IgG was mostly found in maternal and cord blood (Table 2). IgM levels in colostrum and cord blood were similar between the groups. IgG levels in cord blood of diabetic mothers was higher than in maternal and cord blood of normoglycemic mothers. IgA and IgM levels in cord blood were lower than in maternal blood, regardless of glycemic status.

Hyperglycemic and normoglycemic groups had similar spontaneous superoxide release by colostrum mononuclear phagocytes. When exposed to EPEC, superoxide release increased (*P* < 0.05) in both groups. Maternal blood phagocytes present had higher superoxide release when exposed to bacteria. Cord blood phagocytes of hyperglycemic group, independently of bacteria, had higher superoxide release when compared with normoglycemic group (Table 3).

**Figure 1:**
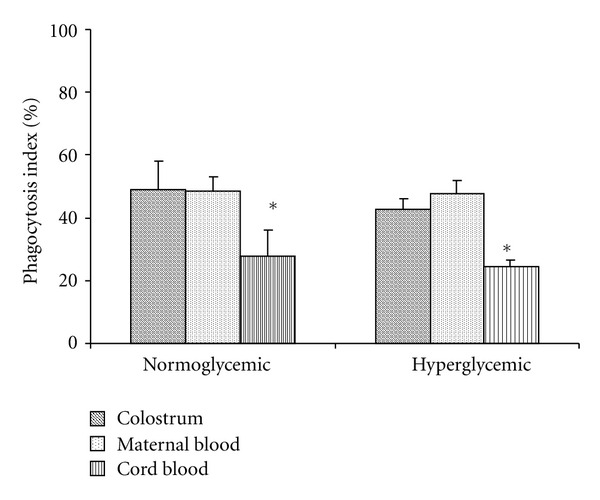
Mean (±SD) phagocytosis index of colostrum, maternal, and cord blood MN cells (*N* = 10) determined using the acridine orange method. *Indicates statistical difference (*P* < 0.05) within normo- and hyperglycemic groups.

**Figure 2:**
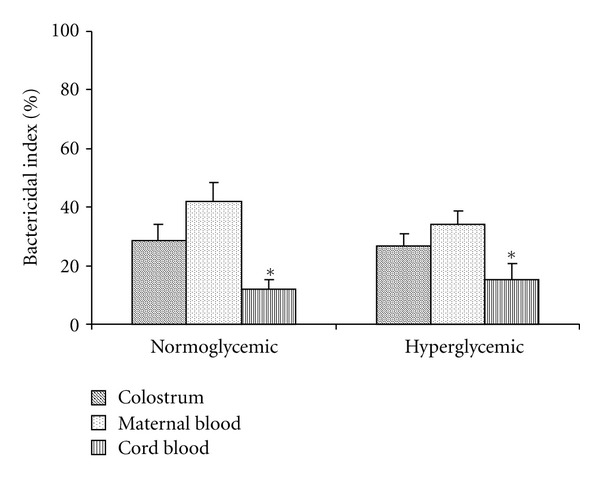
Mean (±SD) bactericidal index of colostrum, maternal, and cord blood MN cells (*N* = 10) determined using the acridine orange method.  *Indicates statistical difference (*P* < 0.05) within normo- and hyperglycemic groups.

MN phagocytes from colostrum and maternal blood exhibited higher phagocytic and microbicidal activity against EPEC than in cord blood, irrespective of the women's glycemic status (Figures 1 and 2).

## 4. Discussion

The present study shows that maternal diabetes altered the transfer of antibodies through the placenta and colostrum. It also describes antibody levels and functional activity of phagocytes in maternal blood, cord blood, and colostrum of hyperglycemic mothers.

Antibody levels in hyperglycemic mothers were significantly lower than in normoglycemic mothers. This result indicates that hyperglycemia changes antibody production in pregnant women, that is, altered levels of plasma glucose may decrease immunoglobulin production. The reduction in immunoreactive protein production may be related to changes in the metabolism of carbohydrates, lipids, and proteins, as well as in various organ systems caused by the hyperglycemic status of pregnant women [12].

IgA and IgM levels were higher in maternal blood than in newborn cord blood from the different groups. Corroborating this result, other authors found higher IgA and IgM concentrations in maternal blood compared to cord blood [20].

Maternal hyperglycemia increased IgG transfer across the placenta since the newborns had higher IgG concentration in cord blood compared to newborns of normoglycemic mothers. The higher plasma glucose level in hyperglycemic women may facilitate IgG transfer via the placenta. During pregnancy, the number of glucose transporters in the placenta of diabetic women increases, enhancing glucose flow to the fetus and possibly related to placental transfer of IgG [21]. 

IgG levels in the hyperglycemic group were lower in maternal blood than in newborn cord blood. This suggests that the reduced IgG production by hyperglycemic mothers enhances immunoglobulin levels in cord blood. Other studies report that the transport efficiency of placental antibody levels increases as maternal antibody levels decrease [22, 23]. In addition, the active transport of IgG occurs when its levels are low in maternal plasma, as observed in mothers and newborns in Africa and Europe [24].

IgA and IgM production by the fetus is active, regardless of its mother's glycemic profile. The concentration of these immunoglobulins in newborn cord blood was not significantly different between hyper- and normoglycemic groups. Other studies performed during embryonic development show that IgA levels are similar in mothers with different glucose profiles [25]. The presence of primary follicles containing B lymphocytes, the precursors of immunoglobulins, can be observed around the 16th week of gestation [17]. Delay in IgA production can compromise the local anti-infective response and predispose newborns to hypersensitivity, and the immaturity of the mucosal immune system of infants may have a critical role in the sensitization of heterologous proteins [26]. 

The morphological differentiation of the elements involved in the immune response begins early in fetal life. The relative and absolute number of B cells in newborns is similar to that of adults. Under normal conditions, the fetus does not produce antibodies and plasma cells are rare. Only in cases of intrauterine infection is the development of germinal centers and differentiation of B lymphocytes observed, with the appearance of plasma cells that start to produce and excrete antibodies [27].

IgM production in the fetus of normal or hyperglycemic mothers begins around the 10th and 15th weeks of gestation. Newborn IgM levels are usually maintained despite the maternal glycemic profile, but they may increase as a result of antigenic stimulation caused by intrauterine infections [28]. 

Newborn antibody production is low compared to adult production and insufficient to produce an effective immune response to microorganisms. Therefore, for several months after birth, the main defense against infections is the passive immunity provided by maternal antibodies through colostrum [2]. In this study, we observed that the immune components of colostrum are modified by hyperglycemia, that is, high plasma glucose levels decrease colostrum IgA and IgG levels.

Since newborns have an immature immune system and the nutritional and immune components of colostrum are important to them [29, 30], soluble and cellular components in human milk, shaped by the mother's previous immune experiences [31], are passively transmitted to newborns, protecting them against infections, modulating their immune system [32] and providing an auxiliary physiological defense mechanism [8]. 

Human colostrum is particularly rich in IgA, which is found in lower amounts in blood plasma. IgA has the ability to block bacterial adherence to epithelial cells [2], acts as an opsonin and plays a protective role against a number of microorganisms [7, 9, 14, 33], avoiding tissue impairment and loss of energy [34].

Despite the alterations observed in colostrum, maternal blood and cord blood antibody levels in the hyperglycemic group, MN phagocyte count and viability were preserved. Earlier studies show that the functional activity of phagocytes was detected in colostrum and blood samples from diabetic patients [35] and from animals with induced diabetes [36, 37]. MN phagocytes play an important role in host defense. They produce phagocytic NADPH oxidase, which forms the superoxide essential to bacterial killing [38, 39] and crucial to the success of immune responses and inflammatory reactions [40]. 

Superoxide release by cord blood MN cells from diabetic mothers in fact increased, independently to EPEC exposure, whereas the maternal blood MN cells increased only in response to EPEC exposure. Excessively high levels of free radicals cause damage to cellular proteins, membrane lipids, and nucleic acids which eventually culminate in triggering of cell death pathways [36, 37, 39]. Various mechanisms have been suggested to contribute to the formation of these reactive oxygen free radicals. Glucose oxidation is believed to be the main source of free radicals [40]. Hyperglycemia has been also found to promote lipid peroxidation by a superoxide-dependent pathway resulting in the generation of free radicals [41, 42]. Another important source of free radicals in diabetes is the interaction of glucose with proteins leading to the formation of various productions that contributed for promotion of free radicals generation and releasing [43].

On the other hand, we observed that MN cells from maternal blood and colostrum had similar phagocytosis and microbicidal activity. The MN cells from cord blood had lower functional activity irrespective of glycemic status. Several studies have shown that the phagocytic and microbicidal activity of colostrum phagocytes [7] is comparable to that of blood phagocytes [29], with similar rates of phagocytosis and bactericidal activity [7, 9].

Interestingly, cord blood phagocytes exposed to EPEC, although exhibited increase superoxide release, this cells present low phagocytosis and bactericidal activity. Some studies report that these cells display low bactericidal activity because they lack nonspecific surface receptors. Hence, the phagocytes do not kill bacteria if they are incubated together [7]. The fact that cord blood phagocytes had lower functional activity reinforces the hypothesis that these cells were immature. 

The rich nutrition transferred passively across the placenta and by breastfeeding in early nutrition can induce permanent beneficial effects on metabolism, growth, immune system, neurodevelopment, and major disease processes [44]. Adequate glycemic control of diabetic mothers is crucial to ensure that the nutritional needs of newborn babies are met and that proper immunity is provided [12]. It can also correct any abnormalities in milk composition [45]. Inadequate glycemic control may result in undesirable consequences such as compromised breastfeeding due to delayed lactogenesis transition from phase I to II [46].

## 5. Conclusion

The study sustains that newborns of diabetic mothers have an immature immune system and is compatible with the immunological profile of newborns of normoglycemic mothers. The hyperglycemia alters IgG transfer across the placenta and decreases immunoglobulin levels in maternal blood and colostrum, given that MN phagocytes exhibit low phagocytic activity against EPEC in cord blood, in both normoglycemic and hyperglycemic groups, and that breastfeeding provides additional protection against intestinal infections in infants.

## Figures and Tables

**Table 1:** Mean (±SD) glucose level, leukocyte count, and viability in colostrum, maternal blood, and cord blood from normoglycemic and hyperglycemic women.

Parameter	Sample	Normoglycemic	Hyperglycemic	Statistical contrast
Glucose level (mg/dL)	Colostrum	76 ± 18.0	148.1 ± 31.8^†^	Colostrum versus blood *F* = 1.77; *P* = 0.57
	Maternal blood	80.95 ± 9.24	111.3 ± 12.7^†^	Normo- versus hyperglycemic *F* = 19.2; *P* = 0.007
	Cord blood	75.3 ± 10.5	95.7 ± 5.7^†^	

Mononuclear phagocytes count (×10^6^ cell/mL)	Colostrum	4.7 ± 0.4	4.5 ± 0.7	Colostrum versus blood *F* = 6.5; *P* = 0.04
	Maternal blood	5.3 ± 0.7	5.1 ± 0.5	Normo- versus hyperglycemic *F* = 2.18; *P* = 0.23
	Cord blood	3.5 ± 0.9*	3.2 ± 1.1*	

Mononuclear phagocytes viability (%)	Colostrum	94 ± 4.5	93 ± 4.2	Colostrum versus blood *F* = 1.85; *P* = 0.71
	Maternal blood	95 ± 5.4	93 ± 4.3	Normo- versus hyperglycemic *F* = 0.8424; *P* = 0.9160
	Cord blood	95 ± 3.4	90 ± 5.7	

*Statistical differences between leukocyte count in cord blood and leukocyte count in maternal blood and colostrum (within a column). ^†^Statistical differences in glucose levels between normoglycemic and hyperglycemic women (in a row).

**Table 2:** Mean (±SD) immunoglobulin level in colostrum, maternal blood, and cord blood from normoglycemic and hyperglycemic women.

Parameter	Sample	Normoglycemic	Hyperglycemic	Statistical contrast
IgG (mg/dL)	Colostrum	120.8 ± 43.7	82.5 ± 27.7^†^	Colostrum versus blood *F* = 78.6; *P* = 0.001
	Maternal blood	2014.6 ± 261.6*	1649.3 ± 207.8^∗†^	Normo- versus hyperglycemic *F* = 16.7; *P* = 0.0003
	Cord blood	1940.4 ± 327.4*	2570.9 ± 466.1^∗#†^	

IgA (mg/dL)	Colostrum	417.8 ± 51.3	291.5 ± 90.3^†^	Colostrum versus blood *F* = 68.87; *P* = 0.0004
	Maternal blood	55.4 ± 7.1*	43.0 ± 5.1*	Normo- versus hyperglycemic *F* = 16.90; *P* = 0.006
	Cord blood	24.4 ± 4.7^∗#^	22.5 ± 8.8^∗#^	

IgM (mg/dL)	Colostrum	36.2 ± 10.5	36.2 ± 8.5	Colostrum versus blood *F* = 15.39; *P* = 0.005
	Maternal blood	52.8 ± 13.8	37.3 ± 6.9^†^	Normo- versus hyperglycemic *F* = 16.81; *P* = 0.02061
	Cord blood	13.5 ± 5.3^∗#^	13.0 ± 7.7^∗#^	

Within a same group (normo- or hyperglycemic), *indicates statistical differences between colostrum and blood and ^#^indicates differences between maternal blood and cord blood immunoglobulin levels. ^†^Indicates statistical differences in immunoglobulin levels between normo- and hyperglycemic women (in a row).

**Table 3:** Superoxide release by colostrum and blood mononuclear phagocytes (mean ± SD, *N* = 10 in each treatment).

Phagocytes	Bacteria	Superoxide release (nmol)		
		Normoglycemic	Hyperglycemic	Statistical contrast
Colostrum	No	1.9 ± 0.4	1.6 ± 0.1	Phagocytes with bacteria versus without *F* = 18.6; *P* = 0.001
	Yes	2.1 ± 0.5	1.5 ± 0.2	

Maternal blood	No	3.5 ± 1.1^#^	4.9 ± 0.9^#^	Colostrum versus blood *F* = 10.6; *P* = 0.002
	Yes	4.4 ± 0.2^∗#^	6.1 ± 0.7^+#^	

Cord blood	No	0.6 ± 0.2^#^	3.8 ± 1.1^+#^	Normo- versus hyperglycemic *F* = 13.7; *P* = 0.03
	Yes	1.2 ± 0.5*	3.9 ± 0.9^+#^	

Colostrum and blood mononuclear cells were preincubated or not with EPEC. *Indicates differences between phagocytes incubated with bacteria and the control (without bacteria) within each group and sample; ^+^indicates intergroup differences within each treatment (with or not bacteria) and sample; ^#^indicates differences between sample (colostrum and blood) within each treatment (with or not bacteria) and group.
